# Identification of MMP28 as a biomarker for the differential diagnosis of idiopathic pulmonary fibrosis

**DOI:** 10.1371/journal.pone.0203779

**Published:** 2018-09-12

**Authors:** Mariel Maldonado, Ivette Buendía-Roldán, Vanesa Vicens-Zygmunt, Lurdes Planas, Maria Molina-Molina, Moisés Selman, Annie Pardo

**Affiliations:** 1 Facultad de Ciencias, Universidad Nacional Autónoma de México, Mexico City, México; 2 Instituto Nacional de Enfermedades Respiratorias, “Ismael Cosio Villegas”, Mexico City, México; 3 Unit of Interstitial Lung Diseases, University Hospital of Bellvitge, Research Institute from Bellvitge (IDIBELL), Barcelona, Spain; 4 Network Centre of Biomedical Research in Respiratory Diseases (CIBERES), Barcelona, Spain; Medizinische Hochschule Hannover, GERMANY

## Abstract

**Background and objective:**

Idiopathic Pulmonary Fibrosis (IPF) is a progressive disease of unknown etiology. The diagnosis is based on the identification of a pattern of usual interstitial pneumonia either by high resolution computed tomography and/or histology. However, a similar pattern can be observed in other fibrotic lung disorders, and precise diagnosis remains challenging. Studies on biomarkers contributing to the differential diagnosis are scanty, and still in an exploratory phase. Our aim was to evaluate matrix metalloproteinase (MMP)-28, which has been implicated in abnormal wound healing, as a biomarker for distinguishing IPF from fibrotic non-IPF patients.

**Methods:**

The cell localization of MMP28 in lungs was examined by immunohistochemistry and its serum concentration was measured by ELISA in two different populations. The derivation cohort included 82 IPF and 69 fibrotic non-IPF patients. The validation cohort involved 42 IPF and 41 fibrotic non-IPF patients.

**Results:**

MMP28 was detected mainly in IPF lungs and localized in epithelial cells. In both cohorts, serum concentrations of MMP28 were significantly higher in IPF versus non-IPF (mostly with lung fibrosis associated to autoimmune diseases and chronic hypersensitivity pneumonitis) and healthy controls (ANOVA, p<0.0001). The AUC of the derivation cohort was 0.718 (95%CI, 0.635–0.800). With a cutoff point of 4.5 ng/mL, OR was 5.32 (95%CI, 2.55–11.46), and sensitivity and specificity of 70.9% and 69% respectively. The AUC of the validation cohort was 0.690 (95%CI, 0.581–0.798), OR 4.57 (95%CI, 1.76–12.04), and sensitivity and specificity of 69.6% and 66.7%. Interestingly, we found that IPF patients with definite UIP pattern on HRCT showed higher serum concentrations of MMP28 than non-IPF patients with the same pattern (7.8±4.4 versus 4.9±4.4; p = 0.04). By contrast, no differences were observed when IPF with possible UIP-pattern were compared (4.7±3.2 versus 3.9±3.0; p = 0.43).

**Conclusion:**

These findings indicate that MMP28 might be a useful biomarker to improve the diagnostic certainty of IPF.

## Introduction

Idiopathic pulmonary fibrosis (IPF) is a chronic, progressive, aging-related lung disease of unknown etiology.[1–3] The prognosis is usually poor, with a median survival time of 2 to 5 years.[1]

The diagnosis of IPF requires the exclusion of recognizable cause of interstitial lung disease (ILD) and identification of a pattern of usual interstitial pneumonia (UIP) either on high-resolution computed tomography (HRCT) or by histology. In the appropriate clinical setting, the presence of UIP pattern on HRCT is sufficient to confirm the diagnosis of IPF. However, the precise diagnosis may be extremely difficult because other chronic fibrotic lung disorders such as ILD associated to connective tissue diseases (primarily rheumatoid arthritis) and chronic hypersensitivity pneumonitis (cHP) may exhibit a UIP-like pattern.[4,5] Unfortunately, biomarkers that may help to distinguish IPF from fibrotic non-IPF ILDs are scanty.

Matrix metalloproteinases (MMPs) are a family of zinc-dependent matrixins that participate in the degradation of the extracellular matrix but also process a variety of mediators such as growth factors, cytokines and chemokines.[6] Importantly, upregulation of several MMPs has been identified in IPF lungs, and two of them, MMP7 and MMP1 have been found increased in sera, and proposed (mainly MMP7) as putative biomarkers for the differential diagnosis.[7–11] Furthermore, it was recently reported that a biomarker index conformed by surfactant protein D (SP-D), MMP7, and osteopontin enhanced diagnostic accuracy in patients with IPF compared with those with non-IPF ILD.[12]

MMP28 is the latest member of the MMPs family and structurally belongs to the MMP19 subfamily,[13] which we revealed as over-expressed in IPF lung epithelium.[8] MMP28 has been reported upregulated in some pathologic conditions such as osteoarthritis,[14] gastric cancer[15] and certain heart conditions such as acute myocardial infarction and unstable angina.[16,17] Recently, we have shown that MMP28 is upregulated in IPF and that MMP28 deficient mice are protected from bleomycin-induced lung fibrosis suggesting a profibrotic role.[18]

Based on these findings we decided to explore the putative role of MMP28 as a diagnostic biomarker in IPF. For this purpose, we examined the lungs by immunohistochemistry and measured this enzyme in blood serum from Mexican patients with IPF, fibrotic ILD associated to autoimmune diseases, chronic hypersensitivity pneumonitis and healthy control subjects (derivation cohort) and in similar groups from Spain (validation cohort).

## Patients and methods

### Study population

Two cohorts of IPF patients were included, one from the Instituto Nacional de Enfermedades Respiratorias, Mexico (INER; n = 82, derivation cohort) and the other from the Unit of Interstitial Lung Diseases of Bellvitge Hospital, Barcelona (n = 42; validation cohort). The diagnosis of IPF was established according to international criteria based on the presence of usual interstitial pneumonia either by HRCT and/or lung morphology.[1] Blood samples were obtained at the time of diagnosis, without previous treatment, and the sera were frozen until use.

In the Mexican cohort, the fibrotic non-IPF group consisted of patients with Rheumatoid Arthritis interstitial lung disease (RA-ILD n = 18), Sjögren Syndrome interstitial lung disease (SS-ILD n = 5), or chronic hypersensitivity pneumonitis (cHP n = 46). Thirty-six age-matched healthy subjects were evaluated as controls.

The Spanish cohort included 41 non-IPF patients (nonspecific interstitial pneumonitis (NSIP), cHP, Scleroderma interstitial lung disease (Scl-ILD) and RA-ILD) and 11 healthy controls.

A multidisciplinary diagnostic team reviewed all final IPF and non-IPF diagnoses. The review boards of both, Instituto Nacional de Enfermedades Respiratorias "Ismael Cosio Villegas", and University Hospital of Bellvitge, approved the study and all patients signed informed consent to participate in the study.

### Immunohistochemistry

We examined the localization of MMP28 in 8 IPF, 5 HP and 5 normal lungs. Immunohistochemical analysis was performed as described.[8] Briefly, formalin-fixed, paraffin-embedded lung tissues were obtained from biopsy or autopsy specimens of individuals with IPF or HP and controls in compliance with institutional review board-approved protocols. Three μm lung sections were deparaffinized, rehydrated and incubated for 30min in 3% H_2_O_2_. After antigen unmasking using citrate buffer, and blocking with 2% of normal sheep serum in PBS, lung sections were incubated for 18 h at 4°C with anti-MMP28 rabbit polyclonal antibody (Novus Biologicals NBP2-17314, 1:100) diluted in PBS with 2% of serum. Sections were then incubated with a secondary biotinylated anti-immunoglobulin antibody followed by horseradish peroxidase-conjugated streptavidin (Biogenex). 3-Amino-9-ethyl-carbazole in acetate buffer containing 0.05% H_2_O_2_ was used as substrate. The sections were counterstained with hematoxylin and mounted with Cristal Mount. The primary antibody was replaced by nonimmune serum for negative control slides. Analysis was performed under a Nikon microscope with NIS-Elements AR software.

### Enzyme-linked-immunosorbent assay

MMP28 concentration in serum was determined by ELISA specific for human MMP28 following the instructions of the manufacturer (SEB999Hu, Clone-Cloud Corporation, USCN, PRC). In addition, in the Mexican cohort, MMP7 was also measured by ELISA (DMP700, R&D). In both cohorts, the same person (MM) did the measurement.

### Statistical analysis

All analyses were performed using Excel 2011 Version 14.7.1, Stata/SE 12.0 for Mac software and Graph Pad Pris 4. To compare IPF versus non-IPF patients, samples were analyzed by U-Mann Whitney. IPF, non-IPF and controls were compared by one-way ANOVA with Dunn's post-tests. p<0.05 was considered of significance. Receiver-operating characteristics (ROC) analysis was used to evaluate sensitivity, specificity and to determine the optimal cut point of MMP28 for differential diagnosis; the area under the curve (AUC) from unadjusted ROC analysis was calculated. We used Odds Ratios (ORs) to determine an association for differential diagnosis between IPF and non-IPF patients. The relationship between patient diagnosis and biomarker was explored using adjusted logistic regression. Demographic [age, sex, smoking status (self-reported and categorized into current, former, or never)] and functional (baseline FVC and DLCO) data were used as potential confounding factors. Additionally, concentrations of MMP28 were correlated with pulmonary functional tests, diffusing capacity of the lung for carbon monoxide (DLCO % predicted) and forced vital capacity (FVC % predicted).

## Results

### Baseline characteristics

This study included 124 IPF patients, 110 non-IPF patients [primarily cHP and fibrotic CTD-ILD (fibrotic NSIP and UIP-like patterns)] and 47 age-matched controls from two cohorts (Mexican controls: 66 ± 8 years; Spanish controls 67±9 years). The derivation cohort was examined in patients living in Mexico City at 2440 meters altitude. The validation cohort was evaluated in Barcelona at sea level. The demographic characteristics and functional abnormalities of the two populations at the time of diagnosis are summarized in **Tables 1 and 2,** and included *in extenso* in **S1 File**. In both Mexican and Spanish cohorts, there was a gender difference (female predominance in the non-IPF cohort) and more smokers in the IPF group. IPF patients from the Mexican cohort were older than non-IPF and exhibited lower oxygen saturation at rest.

**Table 1:** Demographic and functional characteristics, Mexican cohort.

Variable	IPF (n = 82)	Non-IPF (n = 69)	p
Gender (M/F)	66/16	14/55	<0.0001
Age (years)	66 ± 8	57 ± 11	< 0.0001
Smoking status (never/former)	27/55	50/19	<0.001
FVC (% predicted)	74 ± 20	61 ± 28	0.0002
DLCO (% predicted)*	54 ± 25	45 ± 28	0.06
SpO2 rest (%)	88 ± 6	85 ± 8	0.01
SpO2 exercise (%)	82 ± 7	79 ± 7	0.1
Meters (6MWT)	411 ± 156	328 ± 166	0.05

**6MWT**: Six-minute walk test. **FVC:** forced vital capacity, **DLCO:** diffusing capacity of the lung for carbon monoxide.

*Performed in 63 IPF and 44 non-IPF patients at baseline. **SpO2:** oxygen saturation. Tests were performed at 2440mts of altitude. Numbers in age and functional tests are presented as average ± standard deviation.

**Table 2:** Demographic and functional characteristics, Spanish cohort.

Variable	IPF* n = 42	Non-IPF* n = 41	p
Gender (M/F)	36/6	14/27	0.03
Age (years)	67 ± 9	64 ± 11	0.06
Smoking status (never/former)	12/30	24/17	0.006
FVC (% predicted)	77 ± 20	86 ± 23	0.07
DLCO (% predicted)	50 ± 19	60 ± 21	0.02
SpO2 rest (%)	96 ± 2	97 ± 1.4	0.002
SpO2 exercise (%)	89 ± 5	91 ± 6	0.04
Meters (6MWT)	423 ± 75	425 ± 160	0.5

**6MWT:** Six-minute walk test. **FVC:** forced vital capacity. **DLCO:** diffusing capacity of the lung for carbon monoxide. **SpO2:** oxygen saturation.

*Tests were performed at an altitude of 18mts above sea level.

### Immunohistochemical localization of MMP28

**Fig 1:**
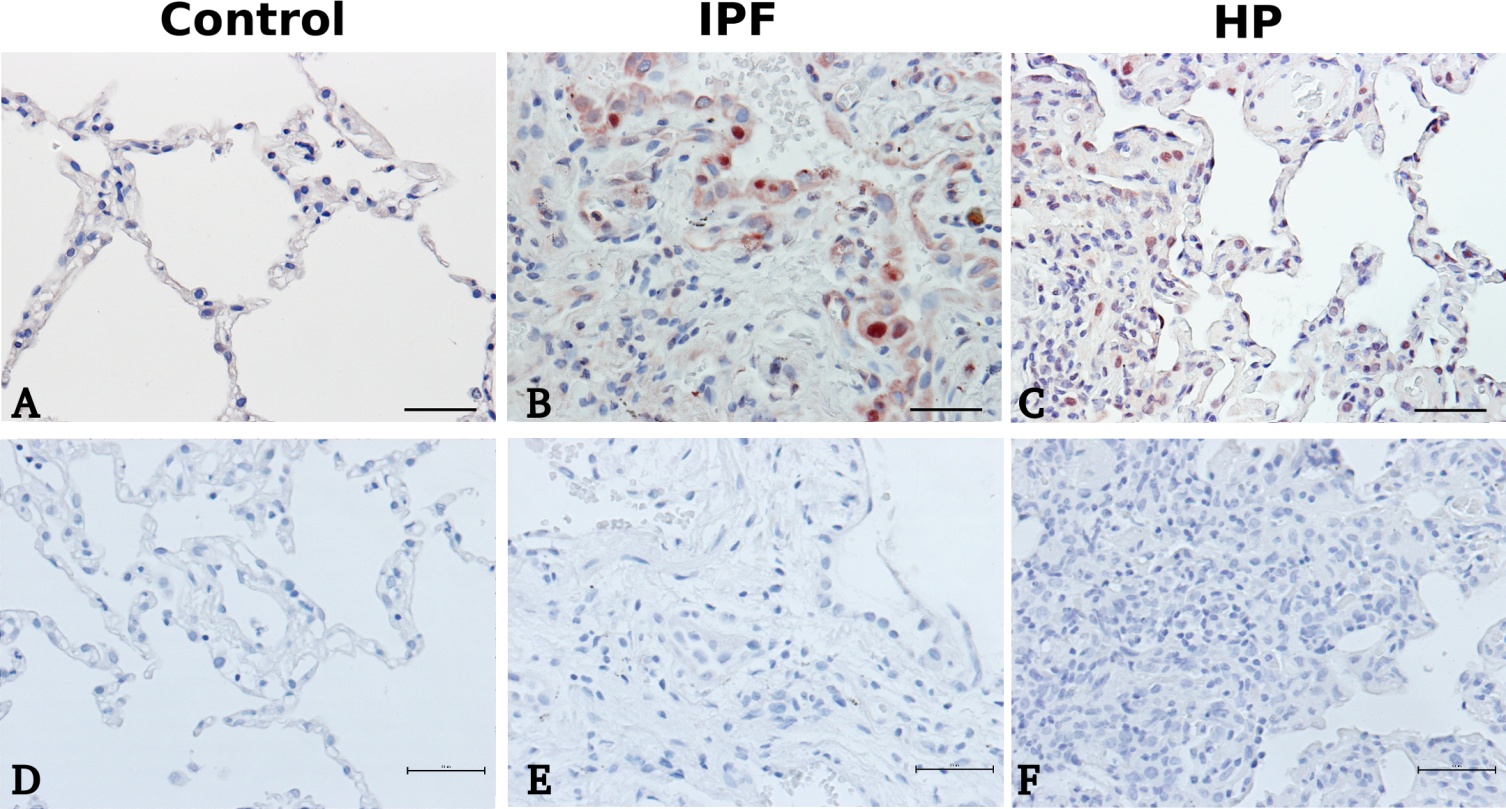
Immunohistochemical localization of MMP28 in idiopathic pulmonary fibrosis, hypersensitivity pneumonitis and control lungs. Representative photomicrographs of immunohistochemical staining performed with specific antibody against MMP28 in control lung tissue sections (panel A), IPF (panel B) and HP (panel C). IHC negative controls were incubated with no primary antibody (panels D, E, F). A-F = 40X magnification (bar = 50μm).

MMP28 cell localization was examined by immunohistochemistry. In IPF lungs, the immunoreactive protein revealed a strong positive labeling mainly in alveolar epithelial cells while a weaker staining was detected in HP, localized mainly in interstitial cells (**Fig 1**). No immunoreactive protein was noticed in the control lungs.

### MMP28 is increased in serum of IPF patients compared with non-IPF patients

**Fig 2:**
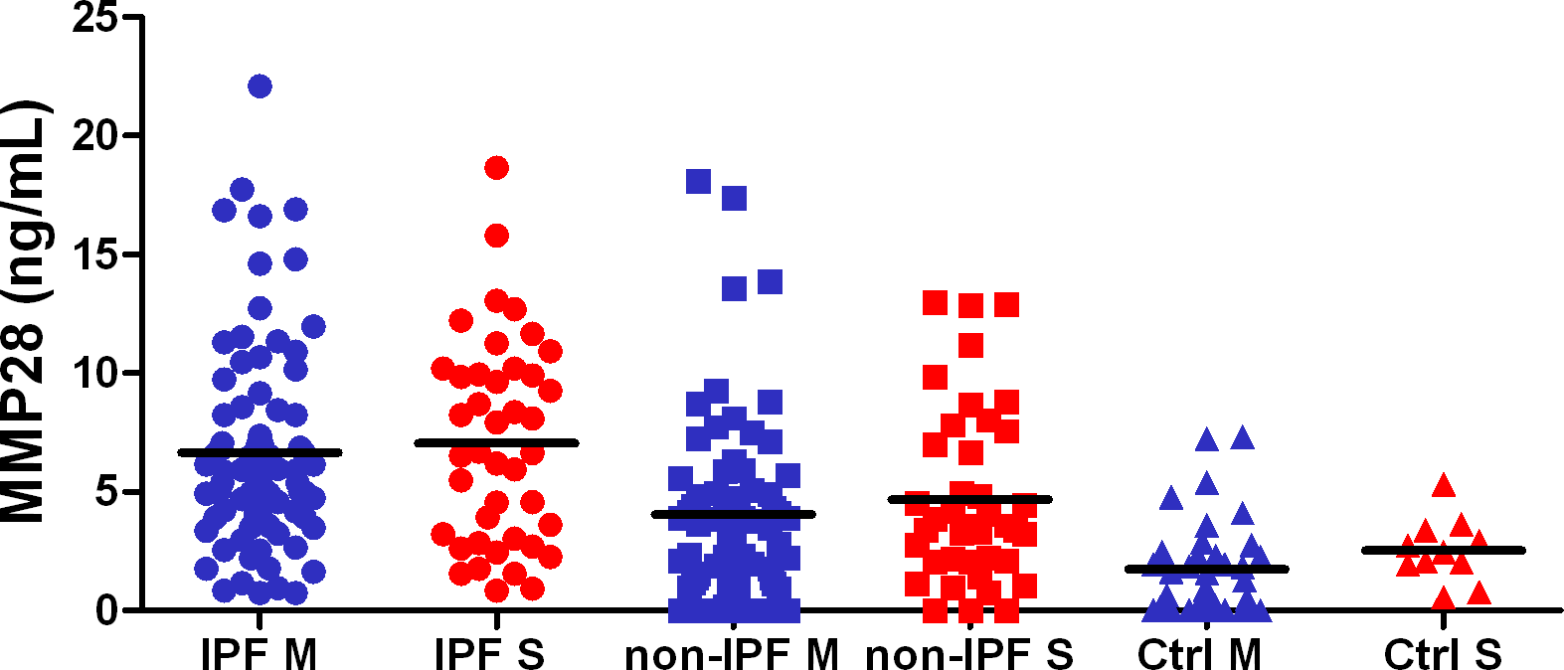
Distribution of MMP28 serum concentrations in IPF, non-IPF and healthy controls. Serum concentrations (ng/ml) of MMP28 in the Mexican cohort (**blue**; ANOVA p<0.0001) and Spanish cohort (**red**; ANOVA p<0.001) are significantly higher in patients with IPF. Averages are represented by horizontal lines.

**Fig 3:**
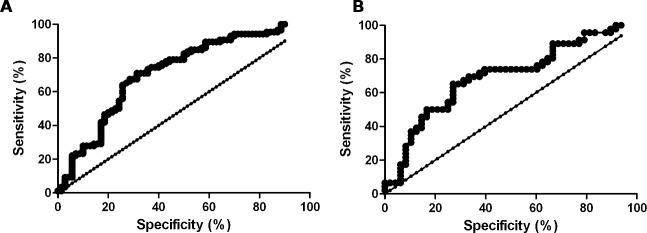
**Receiver operating characteristics curve analysis of serum MMP28 for discriminating IPF from non-IPF disease in Mexican (derivation cohort, A) and Spanish cohort (validation cohort, B)**.

MMP28 concentration in serum from patients and controls of both cohorts was measured by ELISA. In the Mexican (derivation) cohort, MMP28 was found significantly increased in IPF (6.8 ± 4.2 ng/ml) versus fibrotic non-IPF (4.0 ± 3.8 ng/ml) and healthy controls (1.7 ± 1.9 ng/ml) (ANOVA p<0.0001) (**Fig 2, Blue**). Similar results were obtained in the Spanish (validation) cohort IPF (7.1 ± 4.3 ng/ml) versus fibrotic non-IPF (4.7 ± 3.7 ng/ml) and controls (2.5 ± 1.3 ng/ml) (ANOVA p<0.001) (**Fig 2, Red**). In the non-IPF group, no differences between autoimmune diseases and chronic HP were found. The predictive performance of circulating MMP28 for distinguishing patients with IPF from fibrotic non-IPF is shown in **Fig 3**. The AUC of the Mexican cohort was 0.718 (95% CI, 0.635–0.800). With a cutoff point of 4.5 ng/mL of MMP28 in serum, the odds ratio was 5.32 (95%CI, 2.55–11.46), and the sensitivity and specificity were 70.9% and 69% respectively. The positive predictive value was 73% and the negative predictive value was 65%.

The AUC of the cohort from Spain was 0.690 (95% CI, 0.581–0.798). With a cutoff point of 4.5 ng/mL of MMP28 in serum, the odds ratio was 4.57 (95%CI, 1.76–12.04), and the sensitivity and specificity were 69.6% and 66.7% respectively. The positive predictive value was 66% and the negative predictive value was 69%.

In both cohorts, MMP28 continued as a strong predictor of IPF diagnosis after logistic regression analysis with the stepwise method (p = 0.003). Since recent data in mice suggest that MMP28 may promote chronic lung inflammation and tissue remodeling induced by cigarette smoke [19], we compared the serum levels of MMP28 between former- and never- cigarette smokers irrespectively of their diagnosis taking together both cohorts (Mexico and Spain). Our results showed that smokers had higher concentrations of MMP-28 (6.0 ± 4.3 versus non-smoker 5.0 ± 3.9, p = 0.04). However, when we compared the values in IPF and non-IPF by smoking status separately no differences were found: (IPF smokers: 7.1 ± 4.7 ng/ml versus IPF non-smokers: 5.9 ± 3.1 ng/ml; p = 0.17. Non-IPF smokers: 3.9 ± 2.3 ng/ml versus non-IPF non-smoker: 4.5 ± 4.2 ng/ml; p = 0.39).

### IPF patients with UIP pattern on HRCT show higher MMP28 serum concentration compared with non-IPF patients with UIP pattern

We then examined whether MMP28 levels may help to distinguish between definite UIP [1] in the context of IPF versus non-IPF and possible UIP in the context of IPF versus non-IPF (**Table 3**). Taken together both cohorts, we found that IPF patients with definite UIP pattern showed significantly higher serum concentrations of MMP28 compared with non-IPF/UIP pattern (7.8 ± 4.4 versus 4.9 ± 4.4; p = 0.04). By contrast, no differences were observed when IPF with possible UIP-pattern were compared (4.7 ± 3.2 versus 3.9 ± 3.0); p = 0.43).

**Table 3:** High resolution computed tomography findings.

HRCT*	IPF patients (n = 124)	Non-IPF patients (n = 104***)
Definite	84	12
Possible	25	24
Inconsistent**	15	68

*The data represent the HRCT findings of both cohorts.

**IPF patients with inconsistent HRCT were diagnosed by biopsy. All IPF and non-IPF patients were diagnosed by a multidisciplinary diagnostic team.

***HRCT from six non-IPF patients were not available.

### Serum concentrations of MMP7 does not differentiate IPF from non-IPF patients

Since it has been suggested that MMP-7 may also be useful for the differential diagnosis, serum concentration of this enzyme was determined by ELISA in the derivation cohort. Our results showed that this enzyme is similarly increased in IPF (10.24 ±5.7 ng/mL, **n = 64**) and in non-IPF (8.5 ± 5.3 ng/mL, n = **48**), and both are significantly higher than healthy controls (2.1 ± 2.8ng/mL, n = **25**; ANOVA p<0.001). We plotted MMP7 and MMP28 in a scattergram and the correlation coefficient showed that the values were significantly correlated with each other in IPF (rho = 0.5937; p<0.0001; **S1 Fig**). By contrast, only a marginal correlation was found in non-IPF patients (rho = 0.319; p = 0.05).

### Correlation between serum MMP28 and pulmonary function tests

**Fig 4:**
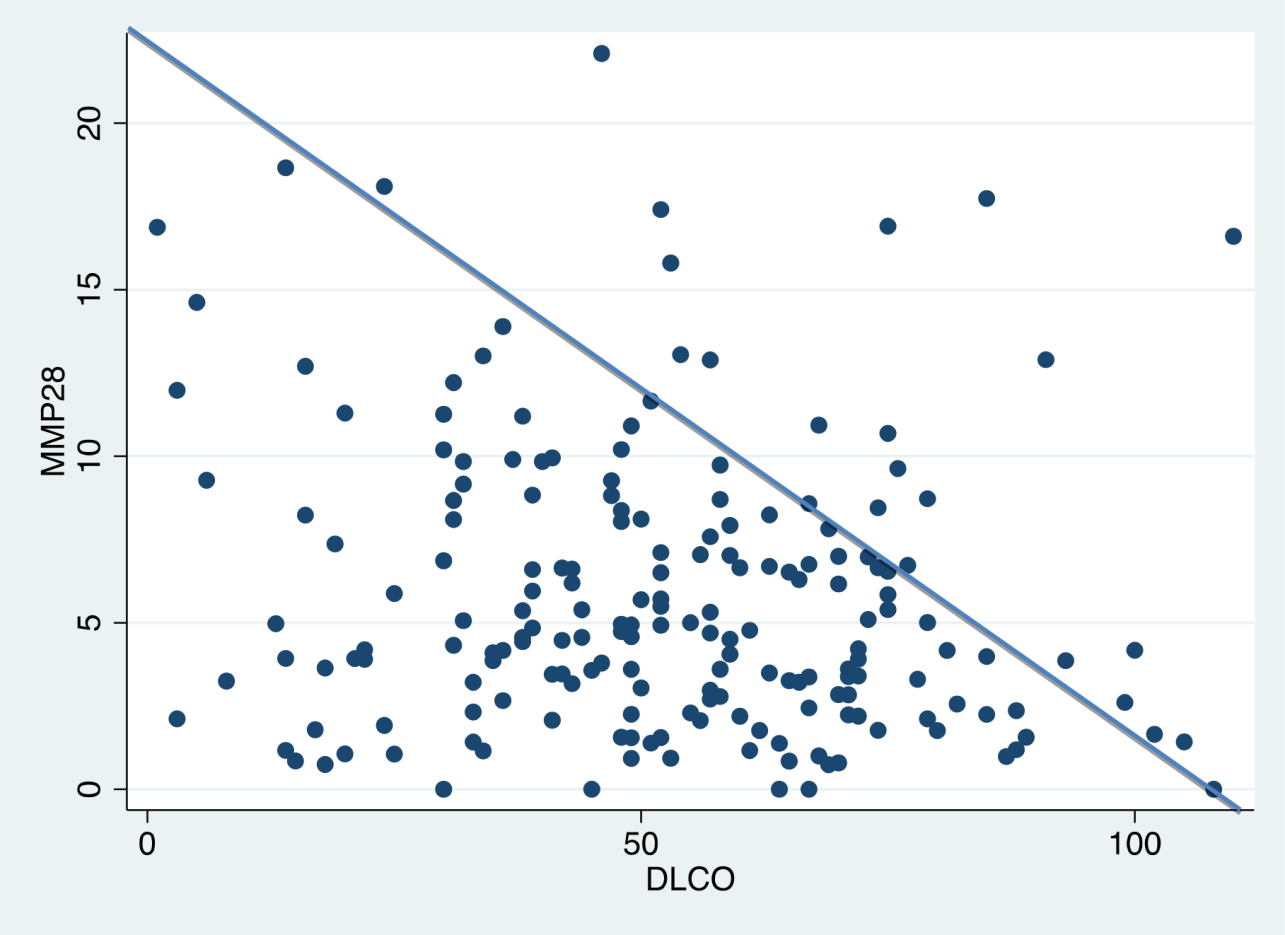
Spearman correlation between diffusing capacity of the lung for carbon monoxide (DLCO) % predicted, and the serum levels of MMP28. The correlation was performed including IPF and non-IPF subjects from both cohorts which had baseline DLCO (n = 190). The superimposed line represents the perfect correlation (Rho = 1) and the circles represent the dispersion of the cases. Rho -0.16; p = 0.02.

The comparison of the concentration levels of MMP28 and diffusing capacity of the lungs for carbon monoxide (DLCO; % predicted) is shown in **Fig 4**. Taken together both cohorts, baseline serum MMP28 concentration demonstrated a marginal but significant negative correlation with DLCO. By contrast, no correlation with forced vital capacity (FVC; % predicted) was found.

## Discussion

IPF is a devastating life-threatening disease that represents one of the major clinical challenges because of its usual progressive nature and because it shares morphological and tomographic UIP-like similarities with other chronic fibrotic lung disorders making difficult the differential diagnosis.

The diagnostic uncertainty represents an important clinical problem since the therapeutic approach is currently completely different. Thus, two drugs nintedanib and pirfenidone were recently approved specifically for the treatment of IPF,[20,21] while corticosteroids and immunosuppressive drugs are indicated in inflammatory and autoimmune-driven ILDs. Moreover, the use of these drugs in IPF is not only useless but dangerous.[22]

In this context, the detection of biomarkers associated with IPF is a promising approach to improve diagnostic accuracy and to overcome the difficulties of current diagnostic strategies.

In the last 10 years, a number of possible biomarkers have been evaluated but most of them appear to predict outcome and have been associated primarily to disease progression and worse survival. These biomarkers include Krebs von den lungen-6 antigen (KL-6), surfactant protein A (SP-A), SP-D, matrix metalloproteinase (MMP)-1, MMP7, lysyl oxidase-like 2 (LOXL2), CC chemokine ligand 18 (CCL18), insulin-like growth factor binding protein-2 (IGFBP-2), heat shock protein 70 (HSP70), periostin, C-X-C motif chemokine 13 (CXCL-13), and neoepitopes from extracellular matrix degradation among others.[23–25] Also, IPF patients displaying an increase of some circulating T-lymphocyte subsets or with an exaggerated shortening leucocyte telomere length have increased risk for clinical deterioration and death.[26,27]

However, biomarkers as a tool for the differential diagnosis of IPF with other fibrotic lung disorders are scanty. To date, MMP7 alone or in combination with other molecules seems to have the potential to discriminate IPF versus non-IPF patients.[10–12] For example, the combination of plasma SP-D, MMP7, and osteopontin was recently demonstrated to enhance diagnostic accuracy to distinguish IPF from other idiopathic interstitial pneumonias, but importantly not with rheumatoid arthritis-associated ILD.[12] In addition, patients with chronic HP, another frequent IPF-mimicking disease, were not included.

In the present study, we explored the role of MMP28 as a putative diagnostic biomarker. This enzyme was selected because as we have recently shown, it is increased in IPF where it may play a profibrotic role increasing the proliferative and migratory phenotype of epithelial cells in a catalytic dependent manner.[18] Likewise, increased expression and release of MMP28 has been reported in hypertrophic scars.[28] Importantly, there is some evidence suggesting that MMP28 induces a coordinated TGF-β-dependent program leading to epithelial to mesenchymal transition, a biological process that has been implicated in the pathogenesis of IPF.[2, 18, 29]

We developed and validated the role of MMP28 as a new biomarker-based for the differential diagnosis of IPF. Specifically, we explored, and we found that the concentration of this metalloprotease in serum is able to distinguish IPF from chronic HP and fibrotic ILD associated to autoimmune diseases. Clinically, both groups are probably the most important targeted cohorts for using diagnostic biomarkers since they are the most likely to show a UIP-like pattern but the therapeutic approach is completely different. This result was demonstrated in two different cohorts of patients indicating that MMP28 levels over 4.5 ng/mL markedly increase the odds of an IPF diagnosis. Taken together both cohorts, ROC curve analysis showed that MMP28 is a useful marker for discriminating IPF from fibrotic non-IPF patients. The AUC of 0.718 in the derivation cohort and 0.690 in the validation cohort further indicated that this enzyme is predictive of IPF with high sensitivity and specificity. Interestingly, when we compared the serum concentration of MMP28 in the context of the radiological pattern, we found that the levels of this enzyme were higher in IPF patients with UIP pattern compared with non-IPF patients with the same pattern, while no differences were observed when we compared possible UIP. This finding suggests that although IPF and non-IPF UIP patterns represent a similar radiologic (and morphologic) phenotype, the different pathogenic mechanisms result in the expression/secretion of different molecules.

As previously mentioned MMP-7 has been suggested to be a useful biomarker to differentiate IPF from non-IPF patients [10–12]. In this context, we also examined the serum concentrations of this enzyme in the derivation cohort. Although, MMP7 was markedly higher in both groups compared with controls, in contrast to our results with MMP28, MMP7 did not discriminate between IPF and non-IPF patients.

In summary, our findings indicate that MMP28 improves diagnostic certainty of IPF and might be included in the diagnostic models of the disease.

## Supporting information

S1 File Demographic and clinical data of each patient from both cohorts. (DOCX)

S1 Fig Scatter plot correlation coefficient between MMP28 and MMP7 in patients with idiopathic pulmonary fibrosis; rho = 0.5937; p<0.0001. (TIF)

## Abbreviations

IPF: Idiopathic Pulmonary Fibrosis
MMP: matrix metalloproteinase
ILD: interstitial lung disease
UIP: usual interstitial pneumonia
HRCT: high-resolution computed tomography
cHP: chronic hypersensitivity pneumonitis
SP-D: surfactant protein D
RA-ILD: Rheumatoid Arthritis interstitial lung disease
ROC: Receiver-operating characteristics
AUC: area under the curve
OR: Odds Ratio
FVC: forced vital capacity
DLCO: diffusing capacity of the lungs for carbon monoxide
