# Supplementary material for: Identification of MMP28 as a biomarker for the differential diagnosis of idiopathic pulmonary fibrosis
Source: PLoS One. 2018 Sep 12;13(9):e0203779. doi: 10.1371/journal.pone.0203779 (PMC6135486; doi:10.1371/journal.pone.0203779)
Supplement: S1 File — (DOCX) [file pone.0203779.s001.docx]

| **Group** | **Age** | **Gender** | **Smoker** | **FVC (%)** | **DLCO(%)** | **Saturation**  **at rest** | **Saturation post exercise** | | **Meters** | **MMP-28** |
| --- | --- | --- | --- | --- | --- | --- | --- | --- | --- | --- |
| **MEXICAN COHORT** | | | | | | | | | |  |
| IPF | 63 | 0 | 0 | 59 | 39 | 95 | 86 | 126 | | 6.59 |
| IPF | 70 | 1 | 0 | 87 | 85 | 85 | 74 | 480 | | 3.98 |
| IPF | 68 | 1 | 1 | 88 | 75 | 92 | 81 | 500 | | 5.39 |
| IPF | 61 | 1 | 1 | 90 | 20 | 93 | 92 | 488 | | 11.29 |
| IPF | 64 | 0 | 1 | 43 | 5 | ND | ND | ND | | 14.62 |
| IPF | 70 | 1 | 1 | 82 | 1 | ND | ND | ND | | 16.87 |
| IPF | 57 | 1 | 1 | 43 | 18 | 60 | ND | ND | | 0.74 |
| IPF | 86 | 1 | 0 | 82 | ND | 89 | 81 | 480 | | 5.00 |
| IPF | 50 | 1 | 1 | 68 | 52 | 87 | 72 | 505 | | 6.50 |
| IPF | 71 | 1 | 1 | 67 | 48 | 89 | ND | ND | | 4.74 |
| IPF | 62 | 1 | 0 | 91 | ND | ND | ND | ND | | 6.16 |
| IPF | 56 | 0 | 1 | 73 | 46 | 95 | 80 | 286 | | 22.09 |
| IPF | 56 | 1 | 0 | 73 | 50 | 89 | 84 | 472 | | 5.69 |
| IPF | 70 | 1 | 0 | 63 | 49 | 94 | 89 | 327 | | 10.91 |
| IPF | 65 | 0 | 0 | 72 | 36 | ND | ND | ND | | 2.66 |
| IPF | 54 | 1 | 0 | 65 | 74 | 91 | 90 | 570 | | 6.65 |
| IPF | 60 | 1 | 1 | 75 | 56 | 89 | ND | ND | | 7.04 |
| IPF | 58 | 1 | 1 | 70 | 72 | ND | ND | ND | | 2.19 |
| IPF | 64 | 1 | 1 | 54 | ND | 80 | ND | ND | | 11.54 |
| IPF | 66 | 1 | 1 | 42 | ND | 85 | ND | ND | | 14.79 |
| IPF | 65 | 1 | 1 | 70 | ND | 81 | ND | ND | | 10.47 |
| IPF | 72 | 1 | 1 | 108 | 67 | 90 | 89 | 265 | | 8.58 |
| IPF | 70 | 1 | 1 | 69 | 52 | ND | ND | ND | | 4.92 |
| IPF | 63 | 1 | 1 | 59 | 57 | 92 | 79 | 218 | | 2.97 |
| IPF | 69 | 1 | 1 | 66 | 70 | ND | ND | ND | | 6.99 |
| IPF | 57 | 1 | 1 | 81 | 67 | 91 | 87 | 537 | | 3.37 |
| IPF | 76 | 1 | 1 | 118 | 110 | 91 | 87 | 571 | | 16.61 |
| IPF | 63 | 0 | 0 | 53 | ND | ND | ND | ND | | 10.15 |
| IPF | 70 | 0 | 1 | 68 | 81 | 94 | 93 | 308 | | 4.17 |
| IPF | 67 | 1 | 1 | 87 | 85 | 93 | 84 | 546 | | 17.74 |
| IPF | 67 | 0 | 0 | 92 | 58 | 93 | 83 | ND | | 9.73 |
| IPF | 52 | 1 | 1 | 56 | ND | 96 | 82 | 381 | | 4.97 |
| IPF | 64 | 1 | 1 | 83 | ND | 89 | ND | ND | | 8.25 |
| IPF | 72 | 1 | 1 | 90 | 77 | 92 | 79 | 529 | | 6.72 |
| IPF | 73 | 1 | 0 | 90 | 70 | ND | ND | ND | | 6.16 |
| IPF | 69 | 1 | 0 | 56 | ND | ND | ND | ND | | 4.98 |
| IPF | 79 | 1 | 1 | ND | ND | 78 | ND | ND | | 11.35 |
| IPF | 70 | 0 | 1 | 66 | 55 | 85 | 74 | 420 | | 5.00 |
| IPF | 71 | 0 | 1 | 81 | 45 | 89 | 83 | 450 | | 3.57 |
| IPF | 76 | 1 | 0 | 97 | ND | 93 | ND | ND | | 0.94 |
| IPF | 78 | 1 | 1 | 39 | ND | 87 | ND | ND | | 4.93 |
| IPF | 66 | 1 | 1 | 42 | ND | 85 | ND | ND | | 12.75 |
| IPF | 58 | 1 | 0 | 94 | 67 | 90 | 82 | ND | | 6.75 |
| IPF | 71 | 1 | 0 | ND | ND | ND | ND | ND | | 3.52 |
| IPF | 79 | 1 | 0 | 105 | 32 | 84 | ND | ND | | 5.06 |
| IPF | 58 | 1 | 1 | 70 | 72 | ND | ND | ND | | 3.91 |
| IPF | 72 | 1 | 1 | 100 | 75 | 91 | 81 | 364 | | 6.54 |
| IPF | 55 | 1 | 1 | 90 | 15 | 92 | 87 | 612 | | 0.85 |
| IPF | 70 | 1 | 1 | 79 | 57 | 90 | 73 | 104 | | 4.69 |
| IPF | 76 | 1 | 1 | 75 | 73 | 87 | 78 | 100 | | 6.97 |
| IPF | 65 | 1 | 1 | 70 | ND | 81 | ND | ND | | 5.92 |
| IPF | 51 | 0 | 1 | 43 | 8 | 73 | ND | ND | | 3.24 |
| IPF | 65 | 1 | 1 | 70 | ND | 81 | ND | ND | | 5.97 |
| IPF | 53 | 0 | 0 | 63 | 43 | 83 | 64 | ND | | 6.61 |
| IPF | 50 | 1 | 0 | ND | ND | ND | ND | ND | | 5.88 |
| IPF | 73 | 1 | 0 | 50 | 69 | 95 | 85 | 424 | | 0.73 |
| IPF | 69 | 0 | 1 | 86 | 49 | 92 | 79 | 140 | | 4.58 |
| IPF | 78 | 1 | 1 | 47 | 63 | 91 | ND | ND | | 3.49 |
| IPF | 69 | 1 | 1 | 120 | 75 | 91 | 85 | 491 | | 5.84 |
| IPF | 72 | 1 | 1 | 81 | ND | 94 | 88 | 638 | | 5.01 |
| IPF | 54 | 1 | 0 | 65 | 74 | 91 | 90 | 570 | | 1.77 |
| IPF | 64 | 0 | 0 | 53 | ND | 86 | ND | ND | | 2.54 |
| IPF | 78 | 0 | 1 | 47 | 63 | 91 | 94 | ND | | 8.24 |
| IPF | 79 | 1 | 0 | 105 | 32 | 84 | ND | ND | | 9.16 |
| IPF | 72 | 1 | 1 | 100 | 75 | 91 | 81 | 364 | | 10.69 |
| IPF | 62 | 1 | 1 | 54 | 22 | 87 | ND | ND | | 4.19 |
| IPF | 63 | 1 | 0 | 53 | 19 | 80 | ND | ND | | 7.36 |
| IPF | 62 | 1 | 1 | 111 | 79 | 93 | ND | 571 | | 5.00 |
| IPF | 69 | 0 | 1 | 120 | 75 | 91 | 85 | 491 | | 16.91 |
| IPF | 54 | 1 | 0 | 65 | 74 | 91 | 90 | 570 | | 8.45 |
| IPF | 65 | 1 | 1 | 76 | 34 | 92 | 78 | 410 | | 1.156 |
| IPF | 64 | 0 | 1 | 96 | 38 | 78 | ND | ND | | 5.36 |
| IPF | 72 | 1 | 0 | 43 | 17 | 85 | ND | ND | | 1.79 |
| IPF | 65 | 1 | 0 | 91 | 72 | 92 | 89 | 390 | | 4.21 |
| IPF | 68 | 1 | 1 | 92 | 14 | 88 | 65 | ND | | 3.93 |
| IPF | 45 | 1 | 1 | 83 | 44 | 91 | 72 | 546 | | 5.39 |
| IPF | 70 | 1 | 1 | 79 | 57 | 90 | 73 | 104 | | 5.31 |
| IPF | 76 | 1 | 1 | 75 | 73 | 87 | 78 | 100 | | 5.09 |
| IPF | 54 | 1 | 1 | 78 | 3 | 84 | ND | ND | | 11.98 |
| IPF | 68 | 1 | 0 | 57 | 82 | 93 | 82 | 468 | | 2.55 |
| IPF | 63 | 1 | 1 | 51 | 30 | 86 | ND | 487 | | 6.86 |
| IPF | 69 | 1 | 1 | 87 | 102 | 94 | 91 | 468 | | 1.65 |
| **SPANISH COHORT** | | | | | | | | | |  |
| IPF | 50 | 1 | 1 | 61 | 21 | 99 | 89 | | 383 | 3.92 |
| IPF | 65 | 1 | 1 | 57 | 31 | 97 | 80 | | 423 | 8.10 |
| IPF | 77 | 1 | 0 | 119 | 65 | 98 | 96 | | 502 | 6.52 |
| IPF | 55 | 0 | 0 | 61 | 48 | 97 | 90 | | 384 | 8.37 |
| IPF | 69 | 1 | 1 | 112 | 51 | 94 | 91 | | 401 | 11.66 |
| IPF | 55 | 1 | 1 | 71 | 47 | 95 | 89 | | 460 | 9.26 |
| IPF | 75 | 1 | 1 | 74 | 99 | 96 | 95 | | 456 | 2.60 |
| IPF | 83 | 1 | 1 | 134 | 68 | 95 | 94 | | 404 | 10.93 |
| IPF | 78 | 0 | 0 | 58 | 37 | 98 | 95 | | 245 | 9.9 |
| IPF | 73 | 1 | 1 | 57 | 42 | 97 | 90 | | 422 | 6.64 |
| IPF | 72 | 1 | 1 | 109 | 57 | 97 | 80 | | 400 | 2.7 |
| IPF | 47 | 1 | 1 | 55 | 16 | 98 | 85 | | 466 | 12.7 |
| IPF | 80 | 0 | 0 | 96 | 43 | 94 | 84 | | 354 | 6.19 |
| IPF | 65 | 1 | 1 | 97 | 70 | 96 | 90 | | 420 | 2.84 |
| IPF | 59 | 0 | 1 | 83 | 53 | 97 | 93 | | 514 | 0.93 |
| IPF | 66 | 0 | 0 | 69 | 30 | 98 | 94 | | 450 | 11.26 |
| IPF | 83 | 1 | 1 | 68 | 58 | 95 | 82 | | 447 | 8.7 |
| IPF | 73 | 1 | 1 | 81 | 30 | ND | ND | | ND | 10.19 |
| IPF | 68 | 1 | 0 | 79 | 63 | 97 | 90 | | 357 | 6.69 |
| IPF | 69 | 1 | 1 | 92 | 54 | 94 | 83 | | 485 | 13.05 |
| IPF | 71 | 1 | 1 | 74 | 59 | 97 | 91 | | 330 | 7.92 |
| IPF | 66 | 1 | 1 | 76 | 53 | 97 | 90 | | 478 | 15.8 |
| IPF | 75 | 1 | 0 | 47 | 32 | 94 | 88 | | 477 | 9.84 |
| IPF | 52 | 1 | 1 | 73 | 52 | 97 | 91 | | 507 | 5.49 |
| IPF | 71 | 1 | 1 | 96 | 62 | 96 | 94 | | 486 | 1.76 |
| IPF | 66 | 0 | 0 | 62 | 48 | 98 | 92 | | 330 | 10.2 |
| IPF | 71 | 1 | 0 | 69 | 65 | 97 | 95 | | 494 | 0.84 |
| IPF | 70 | 1 | 1 | 83 | 76 | 96 | 87 | | 493 | 9.63 |
| IPF | 66 | 1 | 1 | 71 | 89 | 96 | 95 | | 487 | 1.56 |
| IPF | 74 | 1 | 1 | 92 | 50 | 92 | 87 | | 224 | 3.04 |
| IPF | 44 | 1 | 1 | 55 | 16 | 98 | 85 | | 366 | 8.23 |
| IPF | 76 | 1 | 1 | 85 | 67 | 97 | 92 | | 510 | 2.44 |
| IPF | 76 | 1 | 0 | 46 | 41 | 94 | 89 | | 260 | 9.95 |
| IPF | 77 | 1 | 1 | 55 | 14 | 93 | 72 | | 379 | 18.66 |
| IPF | 73 | 1 | 1 | 93 | 59 | 95 | 92 | | 447 | 4.5 |
| IPF | 62 | 1 | 0 | 65 | 39 | 96 | 91 | | 422 | 5.95 |
| IPF | 66 | 1 | 1 | 67 | 31 | 97 | 80 | | 423 | 12.21 |
| IPF | 67 | 1 | 1 | 110 | 66 | 97 | 91 | | 462 | 3.21 |
| IPF | 67 | 1 | 1 | 62 | 38 | 96 | 91 | | 420 | 4.56 |
| IPF | 61 | 1 | 1 | 67 | 71 | 95 | 90 | | 377 | 3.61 |
| IPF | 54 | 1 | 1 | 75 | 49 | 97 | 89 | | 581 | 2.25 |
| IPF | 66 | 1 | 0 | 82 | 48 | 97 | 94 | | 397 | 1.56 |
| Average | 66.5284553 |  |  | 75.39285714 | 52.42857143 | 89.9186047 | 84.1967213 | | 413.827586 |  |
| Standard Deviation | 8.42841136 |  |  | 20.19292408 | 22.70287052 | 6.2482282 | 7.27511666 | | 135.325875 |  |
| **MEXICAN COHORT** | | | | | | | | | |  |
| Non-IPF | 54 | 0 | 0 | 79 | 25 | 77 | 74 | | 350 | 5.87 |
| Non-IPF | 50 | 0 | 1 | ND | ND | ND | ND | | ND | 0 |
| Non-IPF | 35 | 0 | 0 | 71 | 52 | 92 | 89 | | ND | 17.41 |
| Non-IPF | 72 | 0 | 0 | 93 | 36 | 74 | ND | | ND | 4.17 |
| Non-IPF | 49 | 0 | 1 | 44 | 6 | ND | ND | | ND | 9.28 |
| Non-IPF | 72 | 0 | 0 | 32 | ND | 96 | ND | | ND | 5.91 |
| Non-IPF | 55 | 0 | 0 | 55 | ND | 76 | ND | | ND | 7.74 |
| Non-IPF | 76 | 0 | 0 | 68 | 64 | ND | ND | | ND | 1.37 |
| Non-IPF | 72 | 0 | 0 | ND | ND | ND | ND | | ND | 7.52 |
| Non-IPF | 62 | 0 | 0 | 66 | 13 | 82 | ND | | ND | 4.97 |
| Non-IPF | 55 | 0 | 0 | 60 | 3 | 66 | ND | | ND | 2.11 |
| Non-IPF | 66 | 0 | 0 | 104 | 61 | 86 | 72 | | 52 | 4.77 |
| Non-IPF | 59 | 1 | 1 | 50 | 38 | 97 | 95 | | 521 | 4.43 |
| Non-IPF | 55 | 1 | 1 | 58 | 22 | 90 | 77 | | 442 | 3.90 |
| Non-IPF | 64 | 0 | 1 | 69 | 47 | ND | ND | | ND | 8.81 |
| Non-IPF | 57 | 0 | 0 | 77 | 46 | 80 | ND | | ND | 3.79 |
| Non-IPF | 46 | 0 | 0 | 51 | 36 | 91 | 80 | | 305 | 13.89 |
| Non-IPF | 59 | 0 | 0 | 63 | 33 | 93 | 91 | | 445 | 2.32 |
| Non-IPF | 59 | 0 | 1 | 107 | 66 | 91 | 78 | | 484 | 6.30 |
| Non-IPF | 50 | 0 | 0 | ND | ND | ND | ND | | ND | 0 |
| Non-IPF | 36 | 0 | 0 | 45 | 35 | 77 | ND | | ND | 3.86 |
| Non-IPF | 54 | 0 | 0 | 79 | 25 | 77 | 74 | | 350 | 1.05 |
| Non-IPF | 58 | 0 | 0 | 62 | 42 | 83 | ND | | ND | 3.46 |
| Non-IPF | 59 | 0 | 0 | 66 | 49 | 87 | ND | | ND | 4.93 |
| Non-IPF | 35 | 0 | 0 | 30 | 14 | 94 | 83 | | ND | 1.16 |
| Non-IPF | 57 | 0 | 0 | 88 | 88 | 90 | ND | | ND | 2.36 |
| Non-IPF | 60 | 0 | 0 | 39 | ND | ND | ND | | ND | 2.99 |
| Non-IPF | 45 | 0 | 0 | 71 | 85 | 96 | 80 | | 156 | 2.25 |
| Non-IPF | 57 | 0 | 0 | 88 | 88 | 90 | ND | | ND | 1.19 |
| Non-IPF | 62 | 0 | 0 | 75 | 33 | 87 | ND | | ND | 1.41 |
| Non-IPF | 60 | 0 | 1 | 53 | 43 | 67 | ND | | ND | 3.17 |
| Non-IPF | 49 | 0 | 0 | 34 | ND | 85 | ND | | ND | 0 |
| Non-IPF | 62 | 0 | 0 | 36 | ND | 71 | ND | | ND | 0.19 |
| Non-IPF | 58 | 1 | 0 | 39 | 49 | 83 | 71 | | 300 | 0.92 |
| Non-IPF | 38 | 1 | 1 | 32 | 51 | 83 | 80 | | 420 | 1.38 |
| Non-IPF | 65 | 1 | 0 | 62 | 49 | 94 | 75 | | 360 | 1.54 |
| Non-IPF | 44 | 1 | 1 | 79 | 68 | 93 | 90 | | 470 | 1.00 |
| Non-IPF | 26 | 0 | 0 | 46 | ND | 80 | ND | | ND | 1.44 |
| Non-IPF | 50 | 0 | 0 | ND | ND | ND | ND | | ND | 7.25 |
| Non-IPF | 71 | 0 | 0 | 53 | 52 | 81 | ND | | ND | 5.71 |
| Non-IPF | 50 | 0 | 1 | ND | ND | ND | ND | | ND | 4.82 |
| Non-IPF | 65 | 0 | 0 | 107 | 79 | 87 | ND | | ND | 8.72 |
| Non-IPF | 44 | 0 | 0 | 59 | ND | 98 | ND | | ND | 4.15 |
| Non-IPF | 64 | 0 | 0 | 53 | 55 | 94 | 79 | | 182 | 2.29 |
| Non-IPF | 55 | 0 | 0 | 59 | 67 | 93 | 71 | | ND | 0 |
| Non-IPF | 71 | 0 | 0 | 70 | 52 | 81 | ND | | ND | 7.11 |
| Non-IPF | 49 | 0 | 0 | ND | 24 | 86 | 72 | | ND | 18.10 |
| Non-IPF | 50 | 1 | 1 | ND | ND | ND | ND | | ND | 4.33 |
| Non-IPF | 68 | 0 | 0 | 54 | ND | ND | ND | | ND | 1.24 |
| Non-IPF | 55 | 0 | 0 | 73 | 52 | 81 | ND | | ND | 1.55 |
| Non-IPF | 61 | 0 | 1 | 109 | 71 | 90 | 80 | | 390 | 2.83 |
| Non-IPF | 74 | 0 | 0 | 56 | 24 | 84 | ND | | ND | 1.91 |
| Non-IPF | 63 | 0 | 0 | 36 | ND | 77 | ND | | ND | 3.93 |
| Non-IPF | 55 | 1 | 1 | ND | ND | 80 | ND | | ND | 0 |
| Non-IPF | 72 | 0 | 0 | 65 | ND | 88 | ND | | ND | 0 |
| Non-IPF | 41 | 0 | 0 | 38 | ND | 89 | ND | | ND | 0 |
| Non-IPF | 69 | 1 | 0 | 48 | 30 | 93 | 88 | | 490 | 0 |
| Non-IPF | 54 | 0 | 0 | 52 | ND | ND | ND | | ND | 1.97 |
| Non-IPF | 55 | 1 | 1 | 50 | 80 | 90 | 79 | | 250 | 1.76 |
| Non-IPF | 64 | 1 | 1 | 42 | 31 | 83 | ND | | ND | 4.32 |
| Non-IPF | 68 | 1 | 1 | 53 | 35 | 85 | 79 | | ND | 4.09 |
| Non-IPF | 48 | 0 | 1 | 26 | ND | 70 | ND | | ND | 5.09 |
| Non-IPF | 50 | 0 | 0 | ND | ND | ND | ND | | ND | 5.57 |
| Non-IPF | 59 | 0 | 0 | 105 | ND | ND | 79 | | ND | 1.05 |
| Non-IPF | 69 | 0 | 1 | 61 | 18 | 78 | 70 | | 104 | 3.64 |
| Non-IPF | 67 | 0 | 0 | 59 | ND | ND | ND | | ND | 3.91 |
| Non-IPF | 52 | 0 | 1 | 60 | ND | ND | ND | | ND | 4.15 |
| Non-IPF | 74 | 1 | 0 | 82 | ND | ND | ND | | ND | 13.59 |
| Non-IPF | 51 | 1 | 0 | 30 | 50 | 93 | 79 | | 160 | 8.11 |
| **SPANISH COHORT** | | | | | | | | | |  |
| Non-IPF | 61 | 0 | 0 | 85 | 39 | 99 | 96 | | 587 | 8.83 |
| Non-IPF | 42 | 1 | 1 | 62 | 58 | ND | ND | | ND | 3.6 |
| Non-IPF | 68 | 0 | 0 | 106 | 79 | 98 | 96 | | 360 | 2.11 |
| Non-IPF | 78 | 0 | 0 | 54 | 91 | 98 | 75 | | 304 | 12.9 |
| Non-IPF | 37 | 0 | 0 | 95 | 93 | 100 | 96 | | 558 | 3.86 |
| Non-IPF | 74 | 1 | 1 | 77 | 58 | 97 | 96 | | 480 | 2.78 |
| Non-IPF | 64 | 1 | 1 | 97 | 60 | 98 | 93 | | 240 | 6.65 |
| Non-IPF | 52 | 0 | 1 | 91 | 72 | 97 | 94 | | 390 | 3.4 |
| Non-IPF | 79 | 0 | 1 | 113 | 78 | 98 | 96 | | 326 | 3.3 |
| Non-IPF | 80 | 0 | 0 | 98 | 65 | 99 | 90 | | 367 | 3.26 |
| Non-IPF | 55 | 0 | 1 | 116 | 100 | 97 | 96 | | 326 | 4.17 |
| Non-IPF | 71 | 0 | 0 | 158 | 105 | 95 | 95 | | 477 | 1.42 |
| Non-IPF | 60 | 1 | 0 | 68 | 69 | 98 | 88 | | 503 | 7.82 |
| Non-IPF | 67 | 0 | 1 | 85 | 41 | 98 | 94 | | 458 | 3.45 |
| Non-IPF | 77 | 0 | 0 | 93 | 61 | 96 | 87 | | 515 | 1.16 |
| Non-IPF | 54 | 1 | 1 | 89 | 87 | 98 | 95 | | 436 | 0.98 |
| Non-IPF | 74 | 1 | 1 | 66 | 33 | 96 | 79 | | 390 | 3.21 |
| Non-IPF | 68 | 0 | 0 | 99 | 57 | 99 | 96 | | 487 | 7.58 |
| Non-IPF | 65 | 0 | 0 | 98 | 71 | 96 | 90 | | 407 | 2.23 |
| Non-IPF | 46 | 0 | 0 | 104 | 59 | ND | ND | | ND | 4.05 |
| Non-IPF | 68 | 0 | 0 | 69 | 45 | ND | ND | | ND | 0 |
| Non-IPF | 65 | 1 | 1 | 66 | 48 | 95 | 79 | | 476 | 4.95 |
| Non-IPF | 76 | 0 | 0 | 70 | 38 | 94 | 90 | | 310 | 11.2 |
| Non-IPF | 43 | 0 | 0 | 93 | 56 | ND | ND | | ND | 2.06 |
| Non-IPF | 72 | 1 | 1 | 103 | 64 | 96 | 95 | | 380 | 0 |
| Non-IPF | 58 | 1 | 0 | 53 | 57 | 94 | 85 | | 422 | 12.89 |
| Non-IPF | 58 | 1 | 1 | 63 | 44 | 97 | 90 | | 468 | 4.56 |
| Non-IPF | 74 | 0 | 1 | 86 | 39 | 97 | 92 | | 458 | 4.84 |
| Non-IPF | 61 | 0 | 1 | 114 | 40 | 96 | 92 | | 416 | 9.84 |
| Non-IPF | 58 | 0 | 0 | 56 | 34 | ND | ND | | ND | 13.01 |
| Non-IPF | 76 | 0 | 0 | 109 | 108 | ND | ND | | ND | 0 |
| Non-IPF | 65 | 0 | 0 | 58 | 41 | ND | ND | | ND | 2.07 |
| Non-IPF | 59 | 0 | 0 | 103 | 60 | 98 | 97 | | 409 | 2.19 |
| Non-IPF | 72 | 0 | 0 | 97 | 48 | 97 | 87 | | 467 | 8.04 |
| Non-IPF | 79 | 1 | 0 | 116 | 71 | 96 | 96 | | 424 | 3.38 |
| Non-IPF | 66 | 0 | 0 | 60 | 42 | 96 | 90 | | 416 | 4.47 |
| Non-IPF | 72 | 0 | 0 | 65 | 59 | 97 | 93 | | 393 | 7.02 |
| Non-IPF | 54 | 0 | 0 | 43 | 31 | 95 | 86 | | 517 | 8.67 |
| Non-IPF | 57 | 1 | 1 | 60 | 20 | 99 | 83 | | 355 | 1.06 |
| Non-IPF | 66 | 1 | 1 | 86 | 70 | 97 | 96 | | 451 | 0.79 |
| Non-IPF | 44 | 1 | 1 | 87 | 49 | 96 | 93 | | 492 | 3.6 |
| Average | 60.0480769 |  |  | 71.10891089 | 45.15909091 | 85.1730769 | 79.4 | | 327.947368 |  |
| Standard Deviation | 11.3491626 |  |  | 24.85674998 | 21.66225222 | 7.87580349 | 6.75771164 | | 141.784451 |  |

*ND: Not done for severity of disease.

1 = smoker

1 = male
