# Supplementary figures and images for: Identification of MMP28 as a biomarker for the differential diagnosis of idiopathic pulmonary fibrosis
Source: PLoS One. 2018 Sep 12;13(9):e0203779. doi: 10.1371/journal.pone.0203779 (PMC6135486; doi:10.1371/journal.pone.0203779)

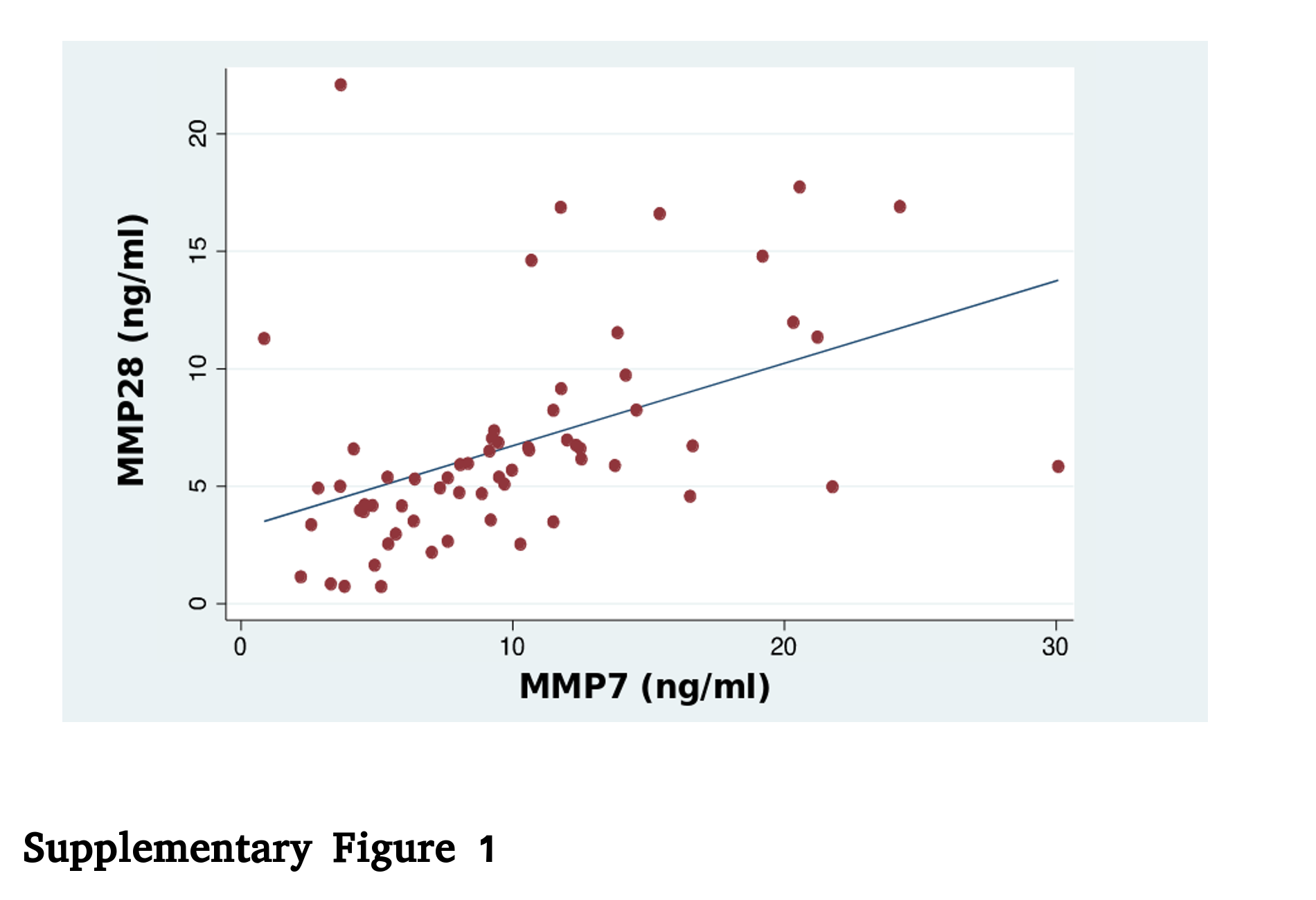

Supplement: S1 Fig — (TIF) [file pone.0203779.s002.TIF]
